# Leptin Contributes to Neuropathic Pain via Extrasynaptic NMDAR-nNOS Activation

**DOI:** 10.1007/s12035-020-02180-1

**Published:** 2020-10-25

**Authors:** Yanling Liang, Yuxin Ma, Jieqin Wang, Lei Nie, Xusheng Hou, Wenyu Wu, Xingmei Zhang, Yinghong Tian

**Affiliations:** 1 Key Laboratory of Mental Health of the Ministry of Education, Guangdong-Hong Kong-Macao Greater Bay Area Center for Brain Science and Brain-Inspired Intelligence, Guangdong Province Key Laboratory of Psychiatric Disorders, Department of Neurobiology, School of Basic Medical Sciences, Southern Medical University, No. 1838 Guangzhou Avenue, Guangzhou, 510515 China; 2 Department of Anatomy, School of Life Sciences and Biopharmaceutics, Guangdong Pharmaceutical University, Guangzhou, 510006 China; 3 Department of Pancreatobiliary Surgery, The First Affiliated Hospital of Sun Yat-sen University, Guangzhou, 510515 China; 4 Department of Anesthesiology, The Third Xiangya Hospital of Central South University, Changsha, 410000 China; 5 Department of Functional Neurosurgery, Zhujiang Hospital, Southern Medical University, Guangzhou, 510515 China; 6 Target and Interventional Therapy Department of Oncology, First People’s Hospital of Foshan, Foshan, 528000 China; 7 Experiment Teaching & Administration Center, School of Basic Medical Sciences, Southern Medical University, No. 1838 Guangzhou Avenue, Guangzhou, 510515 China

**Keywords:** Neuropathic pain, Leptin, NMDAR, Synaptic, Extrasynaptic

## Abstract

Leptin is an adipocytokine that is primarily secreted by white adipose tissue, and it contributes to the pathogenesis of neuropathic pain in collaboration with N-methyl-D-aspartate receptors (NMDARs). Functional NMDARs are a heteromeric complex that primarily comprise two NR1 subunits and two NR2 subunits. NR2A is preferentially located at synaptic sites, and NR2B is enriched at extrasynaptic sites. The roles of synaptic and extrasynaptic NMDARs in the contribution of leptin to neuropathic pain are not clear. The present study examined whether the important role of leptin in neuropathic pain was related to synaptic or extrasynaptic NMDARs. We used a rat model of spared nerve injury (SNI) and demonstrated that the intrathecal administration of the NR2A-selective antagonist NVP-AAM077 and the NR2B-selective antagonist Ro25-6981 prevented and reversed mechanical allodynia following SNI. Administration of exogenous leptin mimicked SNI-induced behavioral allodynia, which was also prevented by NVP-AAM077 and Ro25-6981. Mechanistic studies showed that leptin enhanced NR2B- but not NR2A-mediated currents in spinal lamina II neurons of naïve rats. Leptin also upregulated the expression of NR2B, which was blocked by the NR2B-selective antagonist Ro25-6981, in cultured dorsal root ganglion (DRG) neurons. Leptin enhanced neuronal nitric oxide synthase (nNOS) expression, which was also blocked by Ro25-6981, in cultured DRG cells. However, leptin did not change NR2A expression, and the NR2A-selective antagonist NVP-AAM077 had no effect on leptin-enhanced nNOS expression. Our data suggest an important cellular link between the spinal effects of leptin and the extrasynaptic NMDAR-nNOS-mediated cellular mechanism of neuropathic pain.

## Introduction

Neuropathic pain resulting from injury of the peripheral or central nervous system has several clinical features, including hyperalgesia, allodynia, and spontaneous pain. N-Methyl-D-aspartate receptors (NMDARs) play a crucial role in the mechanisms of peripheral and central sensitization of neuropathic pain [1–4]. There are three main families of NMDAR subunits: NR1, a family of NR2 subunits (NR2A, NR2B, NR2C and NR2D); and two NR3 subunits (NR3A and NR3B). Functional NMDARs are a heteromeric complex and primarily comprise two NR1 subunits and two NR2 subunits. Synaptic and extrasynaptic NMDARs comprise different subtypes. NR2A predominates at synaptic sites, and NR2B is mostly expressed at extrasynaptic sites in mature neurons in the hippocampus and cortex [5, 6], the spinal dorsal horn and dorsal root ganglion (DRG) [7–10]. NR2D is expressed to a lesser extent at extrasynaptic sites in the spinal dorsal horn [9]. NR2C is enriched in the cerebellum and rarely expressed in other brain regions [11, 12].

NR2A and NR2B have different functions. NR2A plays a role in long-term potentiation (LTP), and NR2B is primarily involved in the production of long-term depression (LTD) [13, 14]. In ischemia- and brain trauma-induced neuronal injury, synaptic NMDAR activation promotes neuronal survival, and extrasynaptic NMDAR activation results in neuronal death [15–18]. NR2B plays an important role in the development of neuropathic pain in nerve injury animal models [19–24]. However, whether NR2A is involved in neuropathic pain is controversial [19, 24–27].

Leptin is a 16-kDa adipocytokine that is primarily secreted by white adipose tissue. It is well known for its role in metabolic regulation and obesity, which are mediated via a long-form leptin receptor (Ob-Rb) [28–31]. Leptin and Ob-Rb were recently found in the CNS, which indicates that leptin has a broad role in the regulation of neuronal functions [32, 33]. Recent studies showed that leptin played a significant role in nerve injury-induced neuropathic pain in rats [34, 35]. The peripheral effects of leptin on neuropathic pain following nerve injury were mediated via macrophage stimulation [36], and our previous results indicated that the central effect of leptin was likely related to the activation of NMDARs [34, 35].

Synaptic and extrasynaptic NMDARs contain different subunits, and leptin contributes to the pathogenesis of neuropathic pain via activation of NMDARs. Therefore, the present study further investigated whether leptin contributed to neuropathic pain via synaptic or extrasynaptic NMDARs. Synaptic NMDARs in the spinal cord and DRG primarily comprise NR1/NR2A, and extrasynaptic NMDARs consist of NR1/NR2B and NR1/NR2D [7–10]. However, NR2D does not play a role in the occurrence or development of pain sensation [19, 37], Therefore, we focused on the role of NR2A and NR2B in the contribution of leptin to neuropathic pain.

Neuronal nitric oxide synthase (nNOS) plays an important role in the development and maintenance of neuropathic pain [22, 38–40], and it is a downstream factor of NR2B [19, 22, 41, 42]. The present study also investigated whether leptin contributed to neuropathic pain via synaptic or extrasynaptic NMDAR-nNOS activation. Our results indicated that spinal leptin played an important role in the pathogenesis of neuropathic pain via activation of the extrasynaptic NMDAR-nNOS pathway.

## Materials and Methods

### Experimental Animals

Male Sprague-Dawley rats, 4–6 weeks old, were used for electrophysiological experiments and primary culture of DRG neurons. Rats weighing 250–280 g were used for behavioral experiments. All SD rats were purchased from Southern Medical University Animal Center (Guangzhou, Guangdong, China). The rats were given sufficient food and water and maintained on a 12:12-h light/dark cycle in an SPF animal room at 22–25 °C with 45 ± 10% humidity. All animal protocols were approved by the Animal Experimentation Ethics Committee of Southern Medical University and performed in accordance with the principles of the ‘Administrative Measures for Laboratory Animals of Southern Medical University’ and the ‘Guidelines for Ethical Examination of Animal Experiments of Southern Medical University’.

### Drugs

N-Methyl-D-aspartate (NMDA), glycine, strychnine, bicuculline methochloride, 2,3-dioxo-6-nitro-1,2,3,4-tetrahydrobenzo [f] quinoxaline-7-sulfonamide (NBQX), NVP-AAM077, (+)-5-methyl-10,11-dihydro-5H-dibenzo(a, b)cyclohepten-5,10-imine maleate (MK-801), poly-l-lysine, and cytarabine were purchased from Sigma-Aldrich (St. Louis, MO, USA). Leptin was purchased from Abcam (San Francisco, CA, USA). Ro25-6981 and tetrodotoxin were purchased from Tocris (Ellisville, MI, USA). All drugs were dissolved in water or saline.

### Spared Nerve Injury And Drug Delivery

Spared nerve injury (SNI) was produced according to the method of Decosterd and Woolf [43]. Briefly, rats weighing 280–300 g were anesthetized using pentobarbital sodium (50 mg/kg, intraperitoneal). The skin of the lateral left thigh was incised, and the biceps femoris muscle was separated to expose the sciatic nerve and its three terminal branches: the sural, common peroneal, and tibial nerves. The common peroneal and tibial nerves were tightly ligated, and a 2-mm length segment close to the ligation was removed. The intact sural nerve was untouched. The wound was stitched in two layers.

Intrathecal injections were performed following the procedures described by Wei [44]. A sterile catheter (PE-10 tube) was inserted through the L5/L6 intervertebral space until the tip of the catheter reached spinal lumbar enlargement (approximately 4.5 cm from the incision site for this rat age group). Any rats with hind limb paralysis or paresis after surgery were excluded. Drugs or vehicle were administered using a microsyringe (25 μl) in a 10-μl volume followed by 10 μl of saline to flush the dead space in the catheter. Rats were injected intrathecally once daily for 14 days with leptin (50 μg), Ro25-6981 (20 nmol), NVP-AAM077 (4 nmol), leptin (50 μg) plus Ro25-6981 (20 nmol), leptin (50 μg) plus NVP-AAM077 (4 nmol), or saline. The doses were based on references and our previous results [15, 34, 35, 48].

### Behavioral Test for Allodynia

Rats were habituated to the test environment for 1 h over 2 consecutive days before baseline testing. Mechanical allodynia was examined using von Frey filaments applied to the plantar surface of each hind paw [45]. A positive response was defined as hind paw withdrawal from at least 2 of 5 applications of a von Frey filament. The paw withdrawal threshold (PWT) was determined using the “up-and-down” method [46]. Food intake and body weight were monitored to exclude the effects of these two factors. All behavioral experiments were carried out at 9 AM, and the investigators were blinded to treatment conditions.

### Preparation of Spinal Cord Slices

Lumbar spinal cord slices were prepared from postnatal day 4- to 6-week-old male SD rats. Laminectomy was performed after pentobarbital sodium anesthesia, and a portion of the lumbar spinal cord (L4-L6) was removed and placed in chilled, oxygenated (95% O_2_ + 5% CO_2_) artificial cerebrospinal fluid (ACSF) containing (mM) 125 NaCl, 2.5 KCl, 26 NaHCO_3_, 1.25 NaH_2_PO_4_, 2 CaCl_2_, 2 MgCl_2_, and 25 D-glucose. Transverse slices (350 μm) were cut on a vibratome (Bannockburn, IL, USA). Spinal cord slices were incubated in oxygenated ACSF at 32 °C for 30 min then at room temperature (22–25 °C) for at least 1 h before electrophysiological recording.

### Electrophysiological Recording

Electrophysiological recording was described previously [35]. Briefly, whole-cell patch-clamp recordings were made from lamina II (substantia gelatinosa) neurons of spinal cord slices using glass patch pipettes (1.5 mm outer diameter, 3–5 mΩ resistance). The internal solution contained (mM) 140 K-gluconate, 2 MgCl_2_, 1 CaCl_2_, 11 EGTA, 10 HEPES, 5 Mg-ATP, and 0.5 Na-GTP, pH 7.3. NMDA currents were evoked by ejecting 50 μM NMDA plus 10 μM glycine in Mg^2+^-free ACSF for 30 s at a holding potential of − 70 mV (Stoelting, Kiel, WI, USA). The ejection solution also contained 1 μM strychnine, 10 μM bicuculline, 10 μM NBQX, and 1 μM tetrodotoxin to block glycine receptors, GABA_A_ receptors, AMPA receptors, and voltage-gated sodium channels, respectively. Once the baseline NMDA current was acquired, 100 nM leptin and/or NVP-AAM077 (0.4 μM) and Ro25-6981 (1 μM) were bath-applied for 5 min. All electrophysiological experiments were performed at room temperature (22–25 °C).

### Primary Culture of Rat DRG Neurons

Male SD rats, 4- to 6-weeks-old, were used for the primary culture of DRG neurons [47]. Ganglia from the cervical to the lumbar levels were removed and cut into pieces using a pair of scleral scissors. After enzymatic and mechanical dissociation, the cell suspension was seeded in a poly-l-lysine-coated 96-well plate or 6-well plate. Cultures were maintained at 37 °C in a humidified 95% air/5% CO_2_ incubator (Thermo Scientific, USA) for 3 days in DMEM (Gibco, USA) with 10% serum. The DRG neurons were cultured in medium containing leptin (100 nM), Ro25-6981 (1 μM), NVP-AAM077 (0.4 μM), leptin (100 nM) plus Ro25-6981 (1 μM) or NVP-AAM077 (4 μM) for 3 more days. The doses were based on references and our previous results [15, 34, 35, 48].

### Immunocytochemistry

Cultured DRG neurons in 96-well plates were fixed with 4% paraformaldehyde for 2 h, blocked with bovine serum albumin (3% v/v, Sigma, USA) and Triton-X100 (0.3% v/v, Sigma, USA) for 1.5 h at room temperature and incubated overnight at 4 °C with one of the following primary antibodies: NR2A (Novus, USA) at 1:500, mouse monoclonal; NR2B (Abcam, USA) at 1:1000, rabbit polyclonal; and nNOS (BD Biosciences, USA) at 1:1000, mouse monoclonal. Cells were rinsed with PBS and incubated for 1.5 h at room temperature with an FITC- or cyanine 3-conjugated secondary antibody (1:300; Jackson ImmunoResearch, USA). For controls, primary antibodies were omitted in the process. Four to 6 wells were randomly selected for examination using a fluorescence microscope attached to a CCD spot camera (Olympus IX71, Japan) and processed using Adobe Photoshop v7.

### Western Blot Analysis

Cultured DRG neurons in 6-well plates were lysed in RIPA buffer (Thermo Scientific, USA) supplemented with protease inhibitors (Thermo Scientific, USA). Debris was sedimented via centrifugation at 14,000 g for 10 min at 4 °C, and the supernatant was collected. The protein concentration was detected using a BCA protein assay kit (Thermo Scientific, USA). Proteins were separated using 8% SDS-PAGE gels and transferred to polyvinylidene fluoride (PVDF) membranes (Millipore, Bedford, USA). Membranes were blocked with 5% nonfat dried milk and incubated overnight (4 °C) with anti-NR2A antibody (BOSTER, China: 1:400, rabbit polyclonal), anti-NR2B antibody (Abcam, USA: 1:1000, rabbit polyclonal) [49] and anti-nNOS antibody (BD Biosciences, USA: 1:1000, mouse monoclonal). Membranes were rinsed with TBST and incubated with an HRP-conjugated secondary antibody (1:2000; Sigma-Aldrich, USA) for 1 h at room temperature. After washing with TBST, the protein bands were visualized using an ECL Western blotting kit (Millipore, USA). Densitometry was analyzed using AlphaEaseFC software.

### Statistics

Data from mechanical allodynia (threshold bending force in grams) tests were analyzed using repeated-measure 2-way analysis of variance (ANOVA) as described previously [35]. Testing time points detected overall differences between treatment groups. The data were also examined using 2-way ANOVA across treatment groups to examine overall differences between testing time points. When significant main effects were observed, post hoc Newman–Keuls tests were performed to determine the source(s) of differences. The experimenters were blinded to the treatment conditions. All results are expressed as the means ± SE. Differences were considered statistically significant at the level of *p* = 0.05. For all other experiments, differences were compared using one-way ANOVA followed by post hoc Newman-Keuls tests in SPSS 17.0 (SPSS Inc., Chicago, IL, USA).

## Results

### NR2A and NR2B Antagonists Prevented and Reversed SNI-Induced Allodynia

**Fig. 1:**
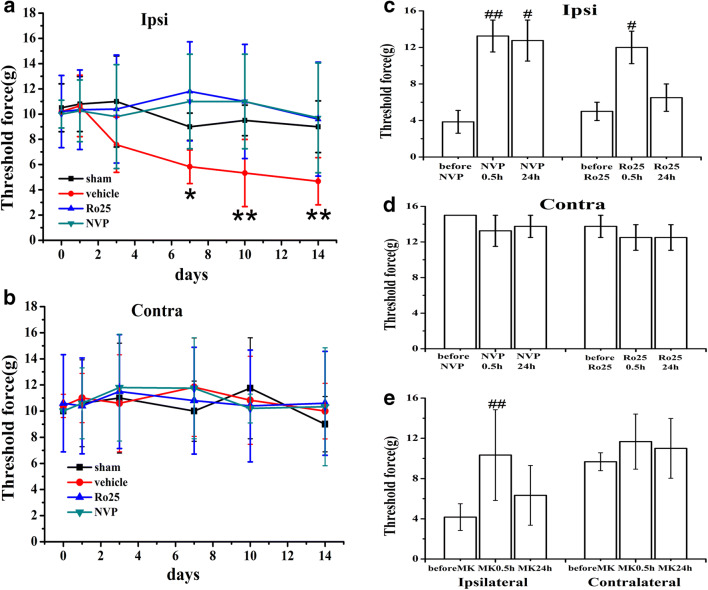
NR2A and NR2B antagonists prevented and reversed mechanical allodynia in rats with SNI. **a** The threshold force of SNI-induced mechanical allodynia on the ipsilateral hind paw was significantly decreased on day 7 and continued until day 14. Intrathecal treatment with the NR2A-selective antagonist NVP-AAM077 (4 nmol) and the NR2B-selective antagonist Ro25-6981 (20 nmol) once daily for 14 days prevented the development of mechanical allodynia on the hind paw ipsilateral to SNI on days 3, 5, 7, 10, and 14. **c** A single intrathecal administration of NVP-AAM077 (4 nmol) and Ro25-6981 (20 nmol) on day 14 attenuated mechanical allodynia at 30 min after the treatment, and the effect of NVP-AAM077 was maintained for 24 h. **b**, **d** NVP-AAM077 and Ro25-6981 did not change the mechanical nociceptive threshold of the contralateral hind paw in the same rat. **e** A single intrathecal administration of 10 nmol MK-801 on day 14 reversed SNI-induced mechanical allodynia 30 min after the treatment. NVP, NVP-AAM077; Ro25, Ro25-6981; MK, MK-801. Data are shown as the means ± SE. *P < 0.05, **P < 0.01 versus vehicle. ^#^*P* < 0.05, ^##^*P* < 0.01 versus before the single intrathecal treatment

To determine whether blockade of synaptic and extrasynaptic NMDARs prevented the development of pain behaviors after SNI, the NR2A-selective antagonist NVP-AAM077 (4 nmol) and the NR2B-selective antagonist Ro25-6981 (20 nmol) were administered intrathecally once daily for 14 days beginning immediately after SNI or sham operation. Behavioral tests on the hind paws were performed 30 min after administration on days 1, 3, 5, 7, 10, and 14. NVP-AAM077 and Ro25-6981 prevented the development of mechanical allodynia of the hind paw ipsilateral to SNI compared to the vehicle group (Fig. 1a*n* = 6; *P* < 0.05). NVP-AAM077 and Ro25-6981 had no effect on mechanical allodynia on the contralateral side in the same rat (Fig. 1b). NVP-AAM077 and Ro25-6981 also reversed established neuropathic pain behaviors in SNI rats. A single intrathecal administration of NVP-AAM077 (4 nmol) and Ro25-6981 (20 nmol) attenuated mechanical allodynia on day 14 after SNI 30 min after treatment, and the effect of NVP-AAM077 was maintained for 24 h (Fig. 1c; *n* = 5; *P* < 0.05). NVP-AAM077 and Ro25-6981 had no effect on the contralateral side (Fig. 1d). The attenuation effect of NVP-AAM077 and Ro25-6981 was the same as treatment with 10 nmol MK-801, which is a noncompetitive NMDAR antagonist (Fig. 1e, *n* = 6). These data demonstrated that NR2A and NR2B antagonists prevented and reversed SNI-induced allodynia.

### NR2A and NR2B Antagonists Prevented Exogenous Leptin-Mimicked SNI-Induced Behavioral Changes

Our previous research found that spinal leptin contributed to the pathogenesis of neuropathic pain, and the central effect of leptin was likely related to the activation of NMDARs [34, 35]. Leptin upregulated the expression of spinal NMDAR subunit NR1 and enhanced NMDAR-induced currents in spinal cord lamina II neurons [34, 35]. The present study further examined whether leptin contributed to the pathogenesis of neuropathic pain by acting on synaptic or extrasynaptic NMDARs.

**Fig. 2:**
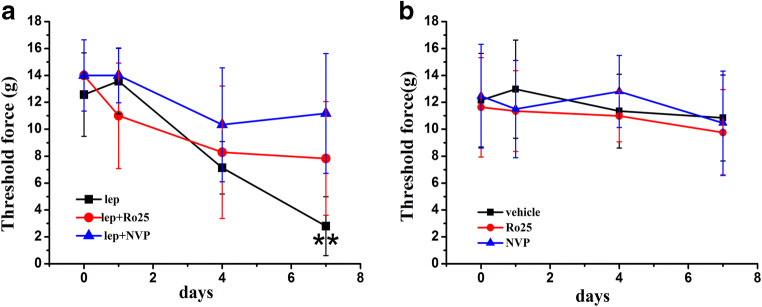
NR2A and NR2B antagonists prevented exogenous leptin-induced mechanical allodynia. **a** Intrathecal leptin (50 μg) treatment in naïve rats, given once daily for 7 days, induced mechanical allodynia on day 7. Coadministration of leptin with 4 nmol NVP-AAM077 or 20 nmol Ro25-6981 attenuated the behavioral changes (*n* = 5). **b** NVP-AAM077 and Ro25-6981 alone did not change the baseline nociceptive threshold (*n* = 6). lep, leptin; NVP, NVP-AAM077; Ro25, Ro25-6981. Data are shown as the means ± SE. **P < 0.01 versus day 0

To determine whether NR2A and NR2B antagonists prevented spinal administration of exogenous leptin-induced mechanical allodynia in naïve rats, different groups of naïve rats received (a) vehicle, (b) 50 μg leptin, (c) 50 μg leptin plus 4 nmol NVP-AAM077, (d) 50 μg leptin plus 20 nmol Ro25-6981, or (e) 4 nmol NVP-AAM077, and (f) 20 nmol Ro25-6981, intrathecally once daily for 7 days. On day 7, intrathecal leptin induced mechanical allodynia in naïve rats similar to SNI rats, and coadministration with 4 nmol NVP-AAM077 or 20 nmol Ro25-6981 prevented this effect (Fig. 2a; *n* = 5; *P* < 0.01). NVP-AAM077 and Ro25-6981 alone did not change the baseline nociceptive threshold (Fig. 2b; *n* = 6). These data showed that NR2A and NR2B antagonists prevented leptin-induced mechanical allodynia.

### Leptin Enhanced NR2B- But Not NR2A-Mediated Currents in Dissociated Lamina II Neurons in Naïve Rats

Our previous results showed that leptin enhanced NMDAR-mediated currents in dissociated substantia gelatinosa (lamina II) neurons of the spinal cord dorsal horn [34, 35]. We chose lamina II neurons because finely myelinated (Aδ) and unmyelinated (C) primary nociceptive afferent fibers project to this spinal region [50, 51]. The present study further examined whether leptin enhanced synaptic or extrasynaptic NMDAR-mediated currents in lamina II neurons in naïve rats.

NMDAR-mediated currents were evoked by ejecting 50 μM NMDA plus 10 μM glycine in Mg^2+^-free ACSF for 30 s at a holding potential of − 70 mV [35]. It was difficult to directly measure synaptic or extrasynaptic NMDAR-mediated currents using whole-cell recording. Therefore, we administered the NR2A-selective antagonist NVP-AAM077 in ACSF to block NR2A-mediated currents and obtain extrasynaptic NMDAR-mediated currents because synaptic NMDARs primarily comprise NR1/NR2A in the spinal cord. Similarly, we obtained synaptic NMDAR-mediated currents via the addition of the NR2B-selective antagonist Ro25-6981 to ACSF to block extrasynaptic NMDAR-mediated currents.

**Fig. 3:**
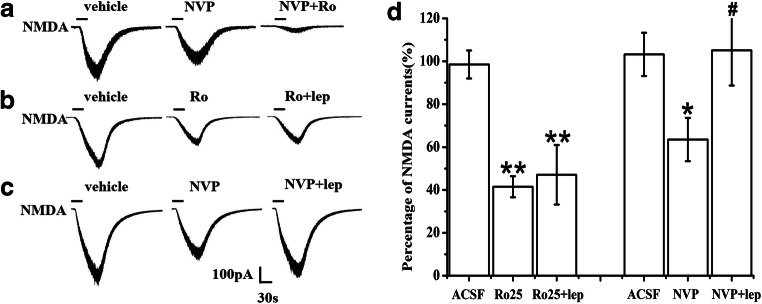
Leptin enhancement of NR2B- but not NR2A-mediated currents in dissociated lamina II neurons in naïve rats. **a** Treatment with the NR2A-selective antagonist NVP-AAM077 (0.4 μM) plus the NR2B-selective antagonist Ro25-6981 (1 μM) blocked NMDAR-mediated currents (*n* = 8). **b** Exposure to leptin (100 nM) for 5 min did not change NMDAR-mediated currents after blockade with Ro25-6981 (1 μM) (*n* = 10). **c** Exposure to leptin (100 nM) for 5 min enhanced NMDAR-mediated currents after inhibition by 0.4 μM NVP-AAM077 (*n* = 9). **d** Histograms showing the effect of leptin on NMDAR-mediated currents after inhibition by NVP-AAM077 or Ro25-6981. Data are shown as the means ± SE. lep, leptin; NVP, NVP-AAM077; Ro, Ro25-6981. *P < 0.05, **P < 0.01 vs. vehicle; ^#^*P* < 0.05 vs NVP

The amplitude of NMDAR-mediated currents ranged from 50 to 500 pA with a mean of 169.47 ± 18.39 pA (Fig. 3). Exposure to 0.4 μM NVP-AAM077 for 5 min reduced less than half of NMDAR-mediated currents. Subsequent exposure to 0.4 μM NVP-AAM077 plus 1 μM Ro25-6981 for 5 min almost completely blocked NMDAR-mediated currents (Fig. 3a). These data suggested that the whole-cell NMDAR-mediated currents comprised NR2A- and NR2B-mediated components.

After treatment with 1 μM Ro25-6981 to block NR2B-mediated currents, the application of 100 nM leptin to ACSF for 5 min did not change the NR2A-mediated currents (Fig. 3b, d). In contrast, the application of 100 nM leptin to ACSF for 5 min significantly enhanced the currents after blockade with 0.4 μM NVP-AAM077 (Fig. 3c, d*P* < 0.05). These results suggested that leptin enhanced extrasynaptic, but not synaptic, NMDAR-mediated currents in dissociated lamina II neurons.

### Leptin Enhanced NR2B, But Not NR2A, Expression in Cultured DRG Neurons

**Fig. 4:**
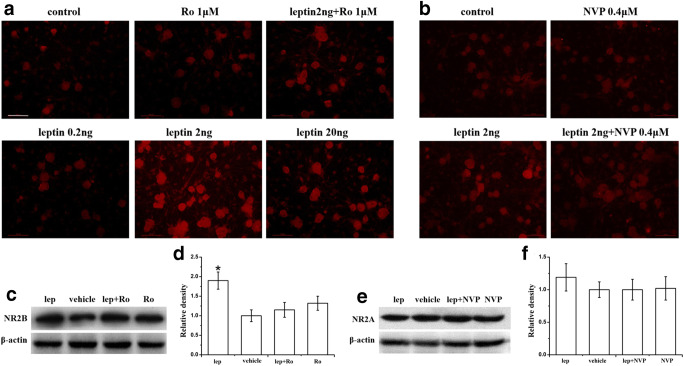
Leptin enhancement of NR2B, but not NR2A, expression in cultured DRG neurons. **a** Immunohistochemistry results showed that administration of leptin in culture medium for 72 h upregulated NR2B expression in a dose-dependent manner (2 ng/ml leptin had the maximal enhancement effect), and cotreatment with 1 μM Ro25-6981 diminished the upregulation. **b** Leptin at 2 ng/ml slightly enhanced NR2A expression, which was attenuated by 0.4 μM NVP-AAM077. **c**–**f** Western blot results showed that administration of leptin (2 ng/ml) to culture medium for 72 h significantly upregulated NR2B expression (**c**, **d**) but not NR2A expression (**e**, **f**) in cultured DRG neurons. The NR2B upregulation was blocked by 1 μM Ro25-6981 (**c**, **d**). Neither 1 μM Ro25-6981 nor 0.4 μM NVP-AAM077 alone changed the baseline expression of NR2B or NR2A. lep, leptin; NVP, NVP-AAM077; Ro, Ro25-6981. *n* = 3. Scale bar, 50 μm. *P < 0.05 vs vehicle

The DRG convey sensory information from the periphery to the CNS and may be damaged in peripheral sensory neuropathic pain [52]. To confirm the relationship of leptin with synaptic or extrasynaptic NMDARs in neuropathic pain, we used an in vitro dissociated DRG neuron culture from adult (postnatal 4- to 6-week-old) naïve rats [47]. DRG neuron culture was established for 72 h, and the culture medium was replaced with a medium containing leptin or vehicle for 72 h [34]. Immunohistochemistry results showed that exposure to exogenous leptin upregulated the expression of NR2B in DRG neuron culture in a dose-dependent manner, with the maximal enhancement occurring at 2 ng/ml leptin (2 ng/ml > 20 ng/ml > 0.2 ng/ml = vehicle. Figure 4a, *n* = 3, *P* < 0.05). This concentration (2 ng/ml) of leptin was used in subsequent culture experiments. Immunohistochemistry results and western blot results showed that the addition of 1 μM Ro25-6981 to the leptin (2 ng/ml) culture medium for 72 h significantly diminished the upregulation of NR2B expression by leptin, whereas 1 μM Ro25-6981 alone did not change the baseline expression of NR2B (Fig. 4a, c, d, *n* = 3). However, 2 ng/ml leptin did not significantly upregulate NR2A expression (Fig. 4b, e, f, *n* = 3). These data showed that leptin significantly upregulated NR2B, but not NR2A, expression in cultured DRG neurons.

### Leptin Enhanced nNOS Expression in Cultured DRG Neurons

**Fig. 5:**
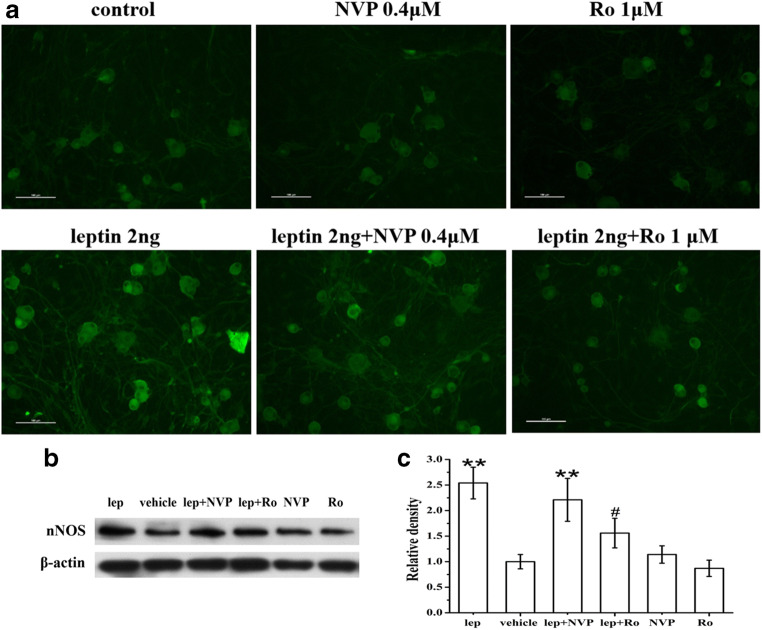
Leptin-mediated enhancement of nNOS expression was blocked by an NR2B antagonist. Immunohistochemistry (**a**) and Western blot (**b** and **c**) results showed that administration of leptin (2 ng/ml) to culture medium for 72 h significantly upregulated nNOS expression in cultured DRG neurons. The upregulation of nNOS expression by leptin was significantly prevented by coapplication of the NR2B antagonist Ro25-6981 (1 μM) and slightly attenuated by the NR2A antagonist NVP-AAM077 (0.4 μM). Ro25-6981 (1 μM) and NVP-AAM077 (0.4 μM) alone did not change baseline nNOS expression. Lep, leptin; NVP, NVP-AAM077; Ro, Ro25-6981. *n* = 3. Scale bar, 50 μm. **P < 0.01 vs vehicle; ^#^*P* < 0.05 vs leptin

To examine the cellular mechanisms underlying the spinal leptin effect on neuropathic pain, we determined whether the expression of nNOS was changed after exogenous leptin treatment because nNOS is a downstream target following NMDAR activation [19, 22, 41, 42]. The inclusion of leptin (2 ng/ml) in culture medium for 72 h significantly upregulated nNOS expression in cultured DRG neurons, as revealed by immunohistochemistry (Fig. 5a) and Western blot (Fig. 5b, c). Coadministration of Ro25-6981 (1 μM), but not NVP-AAM077 (0.4 μM), with leptin effectively prevented the leptin-induced upregulation of nNOS expression. Ro25-6981 (1 μM) and NVP-AAM077 (0.4 μM) alone did not change the baseline nNOS expression. These results suggested that leptin enhanced nNOS expression via NR2B activation.

## Discussion

The present study demonstrated the following results: (1) the intrathecal administration of the NR2A-selective antagonist NVP-AAM077 and the NR2B-selective antagonist Ro25-6981 prevented and reversed SNI-induced allodynia; (2) administration of exogenous leptin mimicked SNI-induced allodynia, which was also prevented by NR2A and NR2B antagonists; (3) leptin enhanced spinal NR2B- but not NR2A-mediated currents in naïve lamina II neurons; and (4) leptin upregulated the expression of NR2B, but not NR2A, and nNOS in cultured DRG cells, which were blocked by the NR2B antagonist Ro25-6981. These data suggested that the contribution of leptin to the pathogenesis of neuropathic pain was related to NR2B-based extrasynaptic NMDARs and activation of nNOS, as the downstream factor of NMDAR. However, the relationship between NR2A-based synaptic NMDARs and the effect of leptin on neuropathic pain needs further investigation.

Leptin is a 16-kDa adipocytokine that is primarily produced by white adipose tissue, and it is well-known for its role in metabolic regulation and obesity [28–31]. Mounting evidence indicates that leptin has broad roles in the regulation of neuronal functions [32, 33]. Leptin was recently demonstrated to contribute to the pathogenesis of neuropathic pain [34–36, 53, 54]. Leptin and leptin receptors (Ob-Rb) are expressed in the spinal cord dorsal horn and DRG and are upregulated after nerve injury [34, 54–57]. Leptin-deficient animals or leptin receptor (Ob-Rb)-deficient animals showed an absence of nerve injury-induced neuropathic pain behaviors, which were reversed by exogenous leptin administration [34, 36, 53, 58]. Chronic administration of exogenous leptin induced thermal hyperalgesia and mechanical allodynia in naïve rats [34, 35]. Treatment with a leptin antibody abolished nerve injury or exogenous leptin administration-induced neuropathic pain behaviors [34, 36]. Our data demonstrated that exogenous leptin administration mimicked SNI-induced mechanical allodynia (Fig. 2a), which further supports the contribution of leptin to neuropathic pain.

NMDARs are one of three ligand-gated ion channels activated by the excitatory transmitter glutamate. Functional NMDARs are a heteromeric complex primarily comprising two NR1 subunits and two NR2 subunits. The different NR2 subunits endow NMDARs with different roles. NR2A predominates at synaptic sites and plays a role in LTP. NR2B is mostly expressed at extrasynaptic sites and is involved in LTD [5, 6, 13, 14]. In ischemia- and brain trauma-induced neuronal injury, NR2A activation promotes neuronal survival, and NR2B activation results in neuronal death [15–18].

NMDARs play a crucial role in the mechanisms of peripheral and central sensitization [1–4]. Our previous studies indicated a possible functional link between leptin and NMDARs in neuropathic pain [34, 35]. The NMDAR antagonist MK-801 attenuated the upregulation of NMDAR subunit NR1 expression and neuropathic pain behavior induced by the spinal administration of exogenous leptin. Leptin enhanced NMDAR-mediated currents in rat spinal cord slices and enhanced spinal NMDA-induced spontaneous BSL behaviors, which are a behavioral indication of spinal excitation.

NR2B plays an important role in the development of neuropathic pain. NR2B is upregulated in the spinal dorsal horn and DRG after peripheral nerve injury [7, 24, 48, 59]. Several lines of evidence showed that phosphorylation of NR2B, especially at Tyr1472, was upregulated after nerve injury, which suggests that spinal NR2B phosphorylation plays a crucial role in central sensitization after nerve injury [19, 37, 60–63]. NR2B special antagonists effectively relieved pain behaviors without causing motor dysfunction [20–23, 48].

The important role of NR2B in the development of neuropathic pain may be due to the impairment of glutamate uptake. Peripheral nerve injury decreased glutamate transporter protein expression and glutamate uptake activity in the spinal dorsal horn [64–66]. Impaired glutamate uptake caused glutamate spillover outside active synapses and increased the open probability of NMDA channels, which led to activation of extrasynaptic NMDA receptors [65, 67, 68]. Nie and Weng demonstrated that blockade of glutamate transporters resulted in an increased proportion of NR2B subunit activation induced by peripheral input, and stronger afferent input further augmented this increase [69]. These data suggested that impairment of glutamate uptake led to the excessive activation of extrasynaptic glutamate receptors, which is a crucial process related to the initiation and maintenance of neuropathic pain.

Our results support a critical relationship between leptin and NR2B-based extrasynaptic NMDARs in neuropathic pain based on the following results: (a) intrathecal administration of the NR2B-selective antagonist Ro25-6981 prevented and reversed SNI-induced allodynia; (b) spinal administration of leptin mimicked SNI-induced allodynia, which was also prevented by Ro25-6981; (c) mechanistic studies showed that leptin upregulated NR2B expression, which was blocked by Ro25-6981, in cultured DRG cells; and (d) leptin enhanced NR2B-mediated currents in spinal cord lamina II neurons. These results indicated that leptin contributed to neuropathic pain via extrasynaptic NMDAR activation. We will further investigate whether leptin directly impairs glutamate uptake and causes glutamate spillover outside of the active synapses, leading to the excessive activation of extrasynaptic NMDA receptors in neuropathic pain.

Whether NR2A is involved in neuropathic pain is controversial. Some studies showed that NR2A expression decreased or did not change in the superficial dorsal horn after nerve injury [7, 24, 70, 71]. Abe et al. found that NR2A-deficient mice developed neuropathic pain similar to wild-type mice after transection of spinal nerve L5 [19]. These data suggest that NR2A is not involved in the process of neuropathic pain. However, other studies demonstrated that the expression level of NR2A increased significantly in the dorsal horn and DRG after nerve injury [25, 27]. Nozaki et al. found that zinc alleviated pain via high-affinity binding to NR2A [26]. These results suggest that the role of NR2A in neuropathic pain is complicated.

The present study found that (a) intrathecal administration of the NR2A-selective antagonist NVP-AAM077 prevented and reversed SNI-induced allodynia, and (b) treatment with NVP-AAM077 also prevented spinal leptin-induced allodynia. However, leptin did not upregulate NR2A expression in cultured DRG cells, and leptin did not enhance NR2A-mediated currents in spinal cord slices. According to these results, whether NR2A is involved in the contribution of leptin to neuropathic pain is not clear.

Three types of NOS, neuronal NOS (nNOS), endothelial NOS (eNOS), and inducible NOS (iNOS), exist in the central nervous system. nNOS plays an important role in the development and maintenance of neuropathic pain. The expression and activation of nNOS in the spinal cord dorsal horn and DRG are increased after nerve injury, and intrathecal injection of a nonselective NOS inhibitor or selective nNOS inhibitor significantly alleviated pain behavior [22, 39, 72, 73]. Overexpression of nNOS decreased the pain threshold in mice with partial sciatic nerve ligation [40], and nerve injury-induced mechanical allodynia was significantly reduced in nNOS gene knockout mice [38].

nNOS is involved in neuropathic pain as a downstream target of NR2B. NR2B is concentrated in the postsynaptic density (PSD), where it integrates dynamically with cytoskeletal proteins and signal transduction factors, such as nNOS, and hence it participates in nociceptive signal transduction [22, 41]. In some pain models, not only nNOS activity and expression was increased, NR2B expression or NR2B phosphorylation at Tyr1472 in the spinal cord dorsal horn or DRG was also increased [19, 39, 42, 74, 75], and NR2B antagonists reversibly reduced nNOS activation to basal levels by [19, 22, 76].

Our data showed that leptin upregulated the expression of NR2B and nNOS in cultured DRG cells, and the NR2B-selective antagonist Ro25-6981 blocked this upregulation (Fig. 4a, Fig. 5). These results and the aforementioned results suggested, for the first time, that the contribution of leptin to the pathogenesis of neuropathic pain was related to the NR2B-based extrasynaptic NMDAR-nNOS pathway. We hypothesized that spinal leptin impairs glutamate uptake after nerve injury. The impairment of glutamate uptake causes glutamate spillover outside of the active synapses, which leads to excessive activation of extrasynaptic NMDAR. Extrasynaptic NMDARs directly interact with nNOS in PSD via the PSD95 scaffold protein, and the activation of extrasynaptic NMDARs increases nNOS activation and nitric oxide (NO) production, which drive nociceptive signal transduction and central sensitization. Our data support extrasynaptic NMDARs as a new target for new pharmacological interventions for neuropathic pain.
